# Functional Prediction of Chronic Kidney Disease Susceptibility Gene PRKAG2 by Comprehensively Bioinformatics Analysis

**DOI:** 10.3389/fgene.2018.00573

**Published:** 2018-12-03

**Authors:** Ermin Wang, Hainan Zhao, Deyan Zhao, Lijing Li, Limin Du

**Affiliations:** ^1^Department of Nephrology, The First Affiliated Hospital, Jinzhou Medical University, Jinzhou, China; ^2^Jinzhou Medical University, Jinzhou, China

**Keywords:** chronic kidney disease, PRKAG2, genome-wide association study, eQTL, gene expression

## Abstract

The genetic predisposition to chronic kidney disease (CKD) has been widely evaluated especially using the genome-wide association studies, which highlighted some novel genetic susceptibility variants in many genes, and estimated glomerular filtration rate to diagnose and stage CKD. Of these variants, rs7805747 in PRKAG2 was identified to be significantly associated with both serum creatinine and CKD with genome wide significance level. Until now, the potential mechanism by which rs7805747 affects CKD risk is still unclear. Here, we performed a functional analysis of rs7805747 variant using multiple bioinformatics software and databases. Using RegulomeDB and HaploReg (version 4.1), rs7805747 was predicated to locate in enhancer histone marks (Liver, Duodenum Mucosa, Fetal Intestine Large, Fetal Intestine Small, and Right Ventricle tissues). Using GWAS analysis in PhenoScanner, we showed that rs7805747 is not only associated with CKD, but also is significantly associated with other diseases or phenotypes. Using metabolite analysis in PhenoScanner, rs7805747 is identified to be significantly associated with not only the serum creatinine, but also with other 16 metabolites. Using eQTL analysis in PhenoScanner, rs7805747 is identified to be significantly associated with gene expression in multiple human tissues and multiple genes including PRKAG2. The gene expression analysis of PRKAG2 using 53 tissues from GTEx RNA-Seq of 8555 samples (570 donors) in GTEx showed that PRKAG2 had the highest median expression in Heart-Atrial Appendage. Using the gene expression profiles in human CKD, we further identified different expression of PRKAG2 gene in CKD cases compared with control samples. In summary, our findings provide new insight into the underlying susceptibility of PRKAG2 gene to CKD.

## Introduction

Chronic kidney disease (CKD) is a major global problem caused by the permanent loss of kidney function, and is also associated with an increased risk for cardiovascular disease (Cusumano and Gonzalez Bedat, 2008; Prodjosudjadi et al., 2009; Chambers et al., 2010; Shinohara, 2010; Sherwood and McCullough, 2016; James et al., 2017; Malhotra et al., 2017; Sinha and Bagga, 2018). The overall prevalence of CKD exceeds 10%, and is approximately 14% in the general population and its incidence is increasing (Almirall, 2016; Hursitoglu, 2016; Mills and He, 2016; Wuttke and Kottgen, 2016; Hedayati et al., 2017; James et al., 2017; Clark et al., 2018). It is reported that up to 20% of CKD cases are caused by genetic forms of renal disease (Almirall, 2016; Hursitoglu, 2016; Mills and He, 2016; Wuttke and Kottgen, 2016; Hedayati et al., 2017; James et al., 2017; Clark et al., 2018). Understanding genetic predisposition to CKD and uncovering underlying pathophysiological mechanisms may contribute to the development of targeted therapies. In recent years, the genetic predisposition to CKD has been widely evaluated especially using the genome-wide association studies (GWAS), which highlighted some novel genetic susceptibility variants in many genes, and estimated glomerular filtration rate to diagnose and stage CKD (Pattaro et al., 2016; Wuttke and Kottgen, 2016).

In these CKD risk genes, a genetic variant rs7805747 in PRKAG2 was identified to be significantly associated with both serum creatinine and CKD with genome wide significance level (Chambers et al., 2010). The rs7805747 (chr7:151407801 for hg19) variant is located in intronic of PRKAG2. PRKAG2 is a protein coding gene. Until now, the potential mechanism by which rs7805747 affects CKD risk is still unclear. It is difficult to identify the function of coding and non-coding genes in molecular wet laboratories. However, computational methods including kinds of bioinformatics software and databases may be useful tools to guide and predict function (Zou et al., 2016; Wan and Zou, 2017; Wan et al., 2017; Wei et al., 2017a,b; He et al., 2018a,b; Jia et al., 2018a,b; Jiang et al., 2018; Zeng et al., 2018). Here, we performed a functional analysis of rs7805747 variant using multiple bioinformatics databases including RegulomeDB (Boyle et al., 2012), HaploReg (version 4.1) (Ward and Kellis, 2016), PhenoScanner (version 1.1) (Staley et al., 2016), and UCSC Genome Browser (Rosenbloom et al., 2015; Tyner et al., 2017; Casper et al., 2018), as did in previous studies (Lu et al., 2011; Rhie et al., 2013; Hazelett et al., 2014; Liu et al., 2016, 2017a,b,c,d,e, 2018b; Guo et al., 2017; Hu et al., 2017a,b; Jiang et al., 2017; Zhang et al., 2018). Meanwhile, we analyzed a whole genome case-control expression profiles in human CKD to investigate whether the susceptibility gene PRKAG2 is differently expressed in CKD cases compared with control samples.

## Materials and Methods

### Regulatory Analysis of rs7805747 Using RegulomeDB

RegulomeDB database could annotate genetic variants with known and predicted regulatory elements in the intergenic regions of the human genome (Boyle et al., 2012). In brief, the known and predicted regulatory DNA elements include regions of DNAase hypersensitivity, binding sites of transcription factors, and promoter regions that have been biochemically characterized to regulation transcription (Boyle et al., 2012). These regulatory element datasets are from Gene Expression Omnibus (GEO), the Encyclopedia of DNA Elements (ENCODE) project, and published literature (Boyle et al., 2012).

### Functional Analysis of rs7805747 Using HaploReg

HaploReg is a tool for exploring annotations of the non-coding variants (Ward and Kellis, 2012, 2016). HaploReg v4 included LD information from the 1000 Genomes Project, chromatin state and protein binding annotation from the Roadmap Epigenomics and ENCODE projects, sequence conservation across mammals, the effect of SNPs on regulatory motifs, and the effect of SNPs on expression from eQTL studies (Ward and Kellis, 2012, 2016). More detailed information is provided in the original studies (Ward and Kellis, 2012, 2016).

### Functional Analysis of rs7805747 Using PhenoScanner

PhenoScanner included publicly available large-scale GWAS summary results, about 3 billion associations and over 10 million unique single nucleotide polymorphisms (SNPs) and a broad range of phenotypes (Staley et al., 2016). The results are aligned across traits to the same effect and non-effect alleles for each SNP (Staley et al., 2016). Here, we performed three kinds of functional analyses including the GWAS, Metabolites, and eQTL analysis options (Staley et al., 2016). To perform a GWAS analysis, the PhenoScanner included 88 GWAS datasets with 76 kinds of diseases or phenotypes (Staley et al., 2016). To perform a Metabolites analysis, PhenoScanner consisted of two metabolomics datasets (Shin et al., 2014; Kettunen et al., 2016). To perform an eQTL analysis, PhenoScanner included several eQTL datasets from eQTL Browser, Geuvadis, GTEx (version 6), MuTHER and bloodeqtlbrowser. More detailed information is provided on the original study (Staley et al., 2016).

### Gene Expression Analysis of PRKAG2 in GTEx

We evaluated the expression of PRKAG2 using the RNA-Seq datasets from the NIH Genotype-Tissue Expression (GTEx) project, which was created to establish a sample and data resource for studies on the relationship between genetic variation and gene expression in multiple human tissues (GTEx Consortium, 2013; Mele et al., 2015). The GTEx project included median gene expression levels in 51 tissues and 2 cell lines (V6, October 2015) (GTEx Consortium, 2013; Mele et al., 2015). This release is based on data from 8555 tissue samples obtained from 570 adult post-mortem individuals (GTEx Consortium, 2013; Carithers et al., 2015; Mele et al., 2015). Here, we used the Genome Browser to evaluate the expression of PRKAG2 in GTEx 53 human tissues (V6, October 2015). The UCSC Genome Browser is a new method to visualize interactions between regions of the genome (Meyer et al., 2013; Karolchik et al., 2014; Rosenbloom et al., 2015; Speir et al., 2016; Tyner et al., 2017; Casper et al., 2018).

### Case-Control Gene Expression Analysis of PRKAG2

We analyzed a whole genome case-control expression profiles in human CKD (Nakagawa et al., 2015). In the original study, a microarray analysis with renal biopsy specimens from CKD patients was conducted to identify the responsible genes associated with tubulointerstitial fibrosis and tubular cell injury in CKD (Nakagawa et al., 2015). This study showed microarray profiles in a total of 61 samples including 53 biopsy specimens of CKD patients, and 8 controls (Nakagawa et al., 2015). Here, we selected the web tool GEO2R to evaluate whether PRKAG2 gene is significantly dysregulated in CKD cases compared with control samples, as did in a recent study (Liu et al., 2018a). The significance level is defined to be *P* < 0.01.

## Results

### Regulatory Analysis of rs7805747 Using RegulomeDB

Using RegulomeDB, the predicted score is 5, which suggested that rs7805747 is likely to affect binding (TF binding) or DNase peak. The predicted binding protein is HNF4A (chr7:151407767-151408030 by ChIP-seq in Caco2 cell type) (Verzi et al., 2010). The histone modification analysis showed that rs7805747 was predicated to locate in enhancer histone marks (Liver, Fetal Intestine Large, Right Ventricle, Duodenum Mucosa, and Fetal Intestine Small). Here, we provided some key information about regulatory analysis in Table 1. More detailed results are described in Supplementary Table 1.

**Table 1:** Key histone modification analysis of rs7805747 using RegulomeDB.

Method	Location	Chromatin state	Tissue
ChromHMM	chr7:151407000..151408400	Enhancers	Fetal intestine large
ChromHMM	chr7:151407200..151408000	Enhancers	Right ventricle
ChromHMM	chr7:151407200..151408200	Enhancers	Duodenum mucosa
ChromHMM	chr7:151407400..151411400	Enhancers	Liver
ChromHMM	chr7:151407000..151408600	Genetic enhancers	Fetal intestine small
ChromHMM	chr7:151406000..151408400	Heterochromatin	H9 derived neuron cultured cells
ChromHMM	chr7:151407400..151408400	Repressed PolyComb	Foreskin fibroblast primary cells skin01
ChromHMM	chr7:151407800..151408400	Repressed PolyComb	Foreskin fibroblast primary cells skin02
ChromHMM	chr7:151405400..151410400	Strong transcription	Monocytes-CD14+ RO01746 primary cells
ChromHMM	chr7:151405600..151408000	Strong transcription	Primary monocytes from peripheral blood


### Functional Analysis of rs7805747 Using HaploReg

Using HaploReg v4, rs7805747 is predicated to locate in enhancer histone marks (Liver, Duodenum Mucosa, Fetal Intestine Large, Fetal Intestine Small, and Right Ventricle tissues), DNase hypersensitivity (Foreskin Melanocyte Primary Cells skin01, Fetal Heart, Fetal Intestine Large, Fetal Intestine Small, Small Intestine), and motifs changed (GATA, HMGN3, Pax-6, and Tgif1) (Ward and Kellis, 2012, 2016).

### Functional Analysis of rs7805747 Using PhenoScanner in GWAS

Using PhenoScanner in GWAS option, we identified 43 significant association results with *P* < 0.01. In addition to the CKD, we found that rs7805747 is also significantly associated with other diseases or phenotypes including Hemoglobin Hb, Hematocrit Hct, Red blood cell count RBC, systolic blood pressure (SBP), Breast cancer, Gout, Hypertension, and Extraversion, as described in Table 2.

**Table 2:** Significant association between rs7805747 and kinds of diseases or phenotypes with *P* ≤ 0.01.

SNP	Trait	PMID	*P*-value	Samples
rs7805747	log(eGFR creatinine in non-diabetics)	26831199	1.50E-29	116602
rs7805747	log(eGFR creatinine)	26831199	8.00E-29	131772
rs7805747	Chronic kidney disease	26831199	2.10E-14	117165
rs7805747	Serum creatinine estimated glomerular filtration rate eGFR without diabetes	22479191	4.00E-14	130600
rs7805747	Serum creatinine	20383146	2.00E-13	90075
rs7805747	Serum creatinine estimated glomerular filtration rate eGFR	22479191	2.70E-13	130600
rs7805747	Serum creatinine estimated glomerular filtration rate eGFR no diabetes	20383146	9.20E-13	90075
rs7805747	Chronic kidney disease	20383146	4.00E-12	NA
rs7805747	Kidney diseases	20383146	4.00E-12	NA
rs7805747	Chronic kidney disease	20383146	4.20E-12	90075
rs7805747	log(eGFR creatinine)	20383146	5.10E-11	67093
rs7805747	Serum creatinine estimated glomerular filtration rate eGFR	20383146	5.10E-11	90075
rs7805747	Chronic kidney disease	22479191	6.00E-10	130600
rs7805747	Serum creatinine estimated glomerular filtration rate eGFR age 65	22479191	7.10E-10	130600
rs7805747	Hemoglobin Hb	23222517	1.80E-09	135367
rs7805747	Serum creatinine estimated glomerular filtration rate eGFR with hypertension	22479191	8.00E-09	130600
rs7805747	Chronic kidney disease	20383146	8.60E-09	62237
rs7805747	Serum creatinine estimated glomerular filtration rate eGFR males	22479191	1.10E-08	130600
rs7805747	Hematocrit Hct	23222517	3.92E-08	135367
rs7805747	Red blood cell count RBC	23222517	9.17E-08	135367
rs7805747	Serum creatinine estimated glomerular filtration rate eGFR females	22479191	9.20E-08	130600
rs7805747	Serum creatinine	20686651	2.20E-07	68439
rs7805747	Serum creatinine estimated glomerular filtration rate eGFR with hypertension	20383146	2.60E-07	90075
rs7805747	Serum cystatin c estimated glomerular filtration rate eGFR	20383146	8.10E-07	90075
rs7805747	Serum creatinine estimated glomerular filtration rate eGFR without hypertension	22479191	8.80E-07	130600
rs7805747	Serum creatinine estimated glomerular filtration rate eGFR age 65	22479191	1.10E-06	130600
rs7805747	log(eGFR cystatin C)	26831199	3.00E-06	32299
rs7805747	Serum creatinine estimated glomerular filtration rate eGFR no hypertension	20383146	4.60E-06	90075
rs7805747	SBP	27618447	6.35E-06	192763
rs7805747	Cystatin C in serum	20383146	1.80E-05	90075
rs7805747	Serum cystatin c estimated glomerular filtration rate eGFR	22479191	1.82E-05	130600
rs7805747	Serum urate	23263486	3.91E-05	94744
rs7805747	Chronic kidney disease severe	22479191	1.00E-04	130600
rs7805747	Breast cancer	23555315	0.00043	14905
rs7805747	log(eGFR cystatin C)	20383146	0.00045	20957
rs7805747	Pulse pressure	27618447	0.0006478	192763
rs7805747	Gout	23263486	0.0007558	69374
rs7805747	Transmission distortion	22377632	0.0009417	4728
rs7805747	HbA1C	20858683	0.001555	46368
rs7805747	Hypertension	27618447	0.003366	183273
rs7805747	Plasma Beta 2 microglobulin levels	23417110	0.0065	6728
rs7805747	Extraversion	26362575	0.006933	63030
rs7805747	Height SDS for females aged 10 years	23449627	0.008512	6967


*eGFR, estimated glomerular filtration rate; SDS, standard deviation scores.*

### Functional Analysis of rs7805747 Using PhenoScanner in eQTL

Using PhenoScanner in eQTL option, we identified 23 significant associations between rs7805747 and gene expression with *P* < 0.01. These findings show that rs7805747 is significantly associated with gene expression in multiple human tissues including brain, cells transformed fibroblasts, colon sigmoid, colon transverse, heart left ventricle, liver, lung, skin, small intestine terminal ileum, stomach, and whole blood, as described in Table 3. These regulated genes include GIMAP1, GIMAP5, AGAP3, KCNH2, TMEM176B, TMEM176A, FASTK, WDR86, NOS3, WDR86-AS1, XRCC2, YBX1P4, SLC4A2, TMUB1, and PRKAG2. Importantly, rs7805747 could significantly regulate PRKAG2 expression in blood with *P* = 3.81E-03 and 8.15E-04.

**Table 3:** Significant association between rs7805747 and the gene expression with *P* ≤ 0.01.

SNP	PMID	Source	Tissue	Gene	Samples	Effect allele	Beta	SE	*P*-value
rs7805747	25954001	GTEx	Brain anterior cingulate cortex ba24	GIMAP1	72	A	–0.474	0.1675	6.68E-03
rs7805747	25954001	GTEx	Brain caudate basal ganglia	GIMAP5	100	A	0.3208	0.07913	1.18E-04
rs7805747	25954001	GTEx	Brain cerebellar hemisphere	AGAP3	89	A	–0.2971	0.07884	3.50E-04
rs7805747	25954001	GTEx	Brain cerebellum	KCNH2	103	A	0.2766	0.07274	2.76E-04
rs7805747	25954001	GTEx	Brain cerebellum	TMEM176B	103	A	0.2503	0.08545	4.41E-03
rs7805747	25954001	GTEx	Brain cortex	TMEM176A	96	A	0.2284	0.06774	1.19E-03
rs7805747	25954001	GTEx	Brain cortex	TMEM176B	96	A	0.196	0.06142	2.08E-03
rs7805747	25954001	GTEx	Brain nucleus accumbens basal ganglia	TMEM176B	93	A	0.2308	0.0691	1.34E-03
rs7805747	25954001	GTEx	Brain nucleus accumbens basal ganglia	FASTK	93	A	–0.1828	0.06627	7.37E-03
rs7805747	25954001	GTEx	Brain putamen basal ganglia	GIMAP5	82	A	0.256	0.0781	1.75E-03
rs7805747	25954001	GTEx	Cells transformed fibroblasts	GIMAP5	272	A	–0.2152	0.07521	4.60E-03
rs7805747	25954001	GTEx	Colon sigmoid	WDR86	124	A	0.1876	0.06838	7.19E-03
rs7805747	25954001	GTEx	Colon transverse	NOS3	169	A	–0.202	0.07399	7.20E-03
rs7805747	25954001	GTEx	Colon transverse	NA	169	A	–0.2624	0.09677	7.59E-03
rs7805747	25954001	GTEx	Heart left ventricle	WDR86-AS1	190	A	0.277	0.1039	8.50E-03
rs7805747	25954001	GTEx	Liver	XRCC2	97	A	–0.3458	0.1297	9.42E-03
rs7805747	25954001	GTEx	Lung	YBX1P4	278	A	0.2894	0.08621	9.19E-04
rs7805747	25954001	GTEx	Skin not sun exposed suprapubic	FASTK	196	A	–0.1002	0.03668	7.01E-03
rs7805747	25954001	GTEx	Small intestine terminal ileum	SLC4A2	77	A	0.3245	0.08101	1.87E-04
rs7805747	25954001	GTEx	Stomach	TMUB1	170	A	0.1734	0.05787	3.26E-03
rs7805747	25954001	GTEx	Whole blood	PRKAG2	338	A	–0.145	0.04971	3.81E-03
rs7805747	22941192	MuTHER	Skin	XRCC2	667	A	0.09756	0.03317	3.30E-03
rs7805747	24013639	Westra H	Peripheral blood	PRKAG2	5311	NA	NA	NA	8.15E-04


*Beta is the regression coefficient based on A allele, which means that A allele regulates increased (Beta > 0) and reduced (Beta < 0) expression of nearby genes.*

### Functional Analysis of rs7805747 Using PhenoScanner in Metabolites

Using PhenoScanner in metabolites option, we identified significant association between rs7805747 and 17 metabolites with *P* < 0.01. These metabolites included Creatinine, Indolelactate, Phenol sulfate, Pseudouridine, Propionylcarnitine, C-glycosyltryptophan, Kynurenine, Myo-inositol, 3-carboxy-4-methyl-5-propyl-2-furanpropanoate (CMPF), 1-palmitoylglycerophosphoethanolamine, Phenyllactate (PLA), Erythronate, N-acetylthreonine, Citrulline, 3-methoxytyrosine, Urate, 2-methylbutyroylcarnitine, as described in Table 4.

**Table 4:** Significant association between rs7805747 and metabolites with *P* ≤ 0.01.

SNP	Trait	PMID	Effect allele	Beta	SE	*P*-value	Samples
rs7805747	Creatinine	27005778	A	0.06017	0.01126	9.76E-08	24806
rs7805747	Indolelactate	24816252	A	0.0131	0.0028	3.77E-06	6939
rs7805747	Phenol sulfate	24816252	A	0.0202	0.0056	3.23E-04	7361
rs7805747	Pseudouridine	24816252	A	0.0065	0.0018	4.24E-04	7336
rs7805747	Propionylcarnitine	24816252	A	0.0093	0.0028	8.46E-04	7364
rs7805747	C-glycosyltryptophan	24816252	A	0.0057	0.0018	1.89E-03	7338
rs7805747	Kynurenine	24816252	A	0.008	0.0027	2.58E-03	7368
rs7805747	Myo-inositol	24816252	A	0.008	0.0027	2.62E-03	7354
rs7805747	3-carboxy-4-methyl-5-propyl-2-furanpropanoate (CMPF)	24816252	A	0.0278	0.0093	2.71E-03	7363
rs7805747	1-palmitoylglycerophosphoethanolamine	24816252	A	0.0081	0.0028	4.48E-03	7317
rs7805747	Phenyllactate (PLA)	24816252	A	0.0081	0.0029	4.87E-03	5702
rs7805747	Erythronate	24816252	A	0.0054	0.0019	5.10E-03	7307
rs7805747	N-acetylthreonine	24816252	A	0.0077	0.0028	6.05E-03	6502
rs7805747	Citrulline	24816252	A	0.0051	0.0019	6.30E-03	7325
rs7805747	3-methoxytyrosine	24816252	A	0.0077	0.0028	6.38E-03	5656
rs7805747	Urate	24816252	A	0.005	0.0018	6.56E-03	7371
rs7805747	2-methylbutyroylcarnitine	24816252	A	0.0076	0.0028	6.76E-03	7007


*Beta is the regression coefficient based on A allele, which means that A allele regulates increased (Beta > 0) and reduced (Beta < 0) metabolites.*

### Gene Expression Analysis of PRKAG2 in GTEx

**FIGURE 1:**
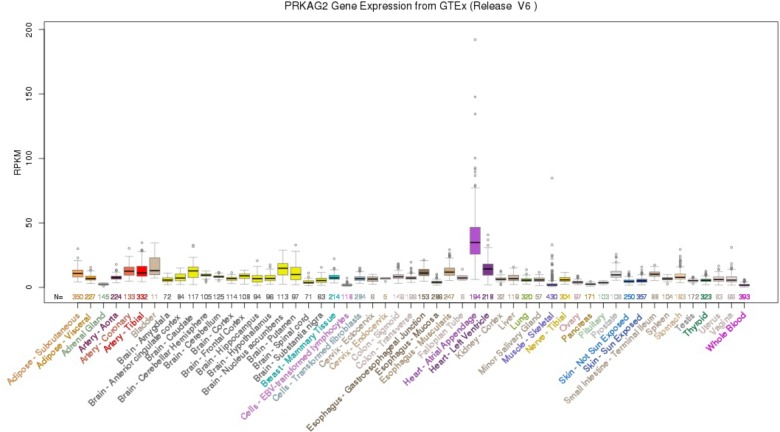
PRKAG2 gene expression in 53 tissues from GTEx RNA-Seq of 8555 samples (570 donors) using UCSC Genome Browser.

Using the UCSC Genome Browser, the results showed that PRKAG2 had the highest median expression: 34.90 RPKM in Heart – Atrial Appendage (Ensembl gene ID: ENSG00000106617.9, Genomic position: hg38 chr7:151556111-151877125). PRKAG2 had the total median expression 412.37 RPKM in all these 53 tissues. Figure 1 provided more detailed information about the PRKAG2 gene expression in 53 tissues from GTEx RNA-Seq of 8555 samples (570 donors).

### Case-Control Gene Expression Analysis of PRKAG2

There are three probes to evaluate the expression of PRKAG2 gene in the gene expression profiles in human CKD including A_24_P384779, A_23_P44366, and A_23_P314760. Each of these three probes represents different regions of PRKAG2 gene. These three probes may have the same or different transcript or isoforms, or extrons. The results showed that all these probes about the PRKAG2 gene is significantly dysregulated in CKD cases compared with control samples including A_24_P384779 [*P* = 1.23E-07 and log2(fold change) = -2.07], and A_23_P44366 [*P* = 4.39E-03 and log2(fold change) = 0.74], and A_23_P314760 [*P* = 1.54E-02 and log2(fold change) = 0.62]. In Table 3, there are 15 unique genes regulated by rs7805747. In addition to PRKAG2, we also evaluated the expression of other 14 genes, as provided in Table 3. The results showed that 4 of other 14 genes including TMUB1, AGAP3, XRCC2, and WDR86-AS1 also had different expression in CKD cases with *P* < 0.01. Table 5 provided the detailed information about 23 probes of 15 genes including PRKAG2. Importantly, the different expression of PRKAG2, TMUB1, AGAP3, and XRCC2 had passed the multiple testing correction threshold 0.01/23 = 0.000435.

**Table 5:** Gene expression analysis of 15 genes regulated by rs7805747 using CKD gene expression dataset.

Probe ID	Gene	Moderated t-statistic	log2(fold change)	*P*-value
A_24_P384779	PRKAG2	–5.955014	–2.0719216	1.23E-07
A_23_P31195	TMUB1	4.8579817	0.9257494	8.08E-06
A_24_P333364	AGAP3	4.7795743	1.1776645	1.08E-05
A_23_P134584	XRCC2	4.0270549	1.5434301	1.53E-04
A_24_P317622	AGAP3	–3.5418815	–1.5220408	7.50E-04
A_32_P138396	WDR86-AS1	3.5246206	1.3570183	7.92E-04
A_23_P44366	PRKAG2	2.9543448	0.7360169	4.39E-03
A_32_P77343	XRCC2	–2.8724894	–1.3978382	5.53E-03
A_23_P314760	PRKAG2	2.4911763	0.6188034	1.54E-02
A_23_P427023	GIMAP1	2.4154446	0.6207405	1.86E-02
A_32_P123479	WDR86	2.2539559	0.8931606	2.77E-02
A_23_P70849	NOS3	1.8409091	0.4171176	7.03E-02
A_23_P253389	SLC4A2	–1.7570194	–0.6506201	8.37E-02
A_23_P31188	GIMAP1	1.6700716	0.3545223	9.98E-02
A_23_P252082	TMEM176A	1.6570106	0.6562679	1.02E-01
A_23_P502930	FASTK	1.6067909	0.3109551	1.13E-01
A_23_P42588	GIMAP5	–1.2244375	–0.337937	2.25E-01
A_23_P377882	KCNH2	–1.1399471	–0.3027131	2.59E-01
A_24_P354998	TMUB1	0.9443127	0.2498472	3.49E-01
A_23_P168403	KCNH2	–0.9265106	–0.4312405	3.58E-01
A_23_P157007	TMEM176B	–0.3719722	–0.1521275	7.11E-01
A_23_P111452	AGAP3	0.3314008	0.1213519	7.41E-01
A_23_P215140	FASTK	0.0048177	0.0008936	9.96E-01


## Discussion

It is reported that PRKAG2 could encode the gamma2-subunit isoform of 5′-AMP-activated protein kinase (AMPK) (Ahmad et al., 2005; Banerjee et al., 2007; Folmes et al., 2009; Kim et al., 2012; Thorn et al., 2013; Zhang et al., 2013; Hinson et al., 2016, 2017). AMPK is a metabolic enzyme, which plays important roles in regulating of energy metabolism in response to cellular stress. AMPK has been identified to be a regulator of metabolism, survival, and fibrosis, by a recent integrative analysis of PRKAG2 cardiomyopathy iPS and microtissue models (Hinson et al., 2016). In addition, mutations in PRKAG2 have been identified to be associated with hypertrophic cardiomyopathy (Xu et al., 2017).

Over the past decade, GWAS have considerably improved our understanding of the genetic basis of kidney function and disease (Wuttke and Kottgen, 2016). A SNP rs7805747 identified by CKD GWAS lies upstream of PRKAG2. Here, we performed a comprehensively functional analysis of this variant using multiple bioinformatics databases including RegulomeDB (Boyle et al., 2012), HaploReg (version 4.1) (Ward and Kellis, 2016), and PhenoScanner (version 1.1) (Staley et al., 2016). Using RegulomeDB, rs7805747 is predicted to affect HNF4A binding or DNase peak. Using RegulomeDB, the predicted score is 5, which suggested that rs7805747 is likely to affect binding (TF binding) or DNase peak. The predicted binding protein is HNF4A (chr7:151407767-151408030 by ChIP-seq in Caco2 cell type). In addition, rs7805747 was predicated to locate in enhancer histone marks (Liver, Fetal Intestine Large, Right Ventricle, Duodenum Mucosa, and Fetal Intestine Small). Using HaploReg (version 4.1), we identified rs7805747 to be associated with enhancer histone marks, DNase hypersensitivity, and motifs changed. In HaploReg (version 4.1), rs7805747 was also predicated to locate in enhancer histone marks (Liver, Duodenum Mucosa, Fetal Intestine Large, Fetal Intestine Small, and Right Ventricle tissues). Hence, the findings in HaploReg (version 4.1) were consistent with RegulomeDB.

Using PhenoScanner in GWAS option, we showed that rs7805747 is not only associated with CKD, but also is significantly associated with other diseases or phenotypes including Hemoglobin Hb, Hematocrit Hct, Red blood cell count RBC, SBP, Breast cancer, Gout, Hypertension, and Extraversion.

Using PhenoScanner in eQTL option, rs7805747 is identified to be significantly associated with gene expression in multiple human tissues and multiple genes including PRKAG2. Previous study has reported rs7805747 to be associated with serum creatinine and CKD (Chambers et al., 2010). Using PhenoScanner in metabolites option, rs7805747 is identified to be significantly associated with not only the serum creatinine, but also with other 16 metabolites, as described in Table 4.

The gene expression analysis of PRKAG2 using 53 tissues from GTEx RNA-Seq of 8555 samples (570 donors) in GTEx showed that PRKAG2 had the highest median expression in Heart – Atrial Appendage. Using the gene expression profiles in human CKD, we further identified different expression of PRKAG2 gene in CKD cases compared with control samples. All these findings indicate that rs7805747 is associated with CKD risk, PRKAG2 gene expression, and 17 metabolites. Meanwhile, gene expression analysis further showed that CKD cases had different expression of PRKAG2 gene. In summary, our findings provide new insight into the underlying susceptibility of PRKAG2 gene to CKD.

## Author Contributions

EW conceived and initiated the project and performed the functional analysis. EW, HZ, DZ, LL, and LD wrote the manuscript. All authors reviewed the manuscript and contributed to the final manuscript.

## Conflict of Interest Statement

The authors declare that the research was conducted in the absence of any commercial or financial relationships that could be construed as a potential conflict of interest.
