# Supplementary material for: Functional Prediction of Chronic Kidney Disease Susceptibility Gene PRKAG2 by Comprehensively Bioinformatics Analysis
Source: Front Genet. 2018 Dec 3;9:573. doi: 10.3389/fgene.2018.00573 (PMC6287114; doi:10.3389/fgene.2018.00573)
Supplement: Supplementary file 1 [file Table_1.DOC]

Supplementary Table 1, histone modification analysis of rs7805747 using RegulomeDB

| Method | Location | Chromatin state | Tissue |
| --- | --- | --- | --- |
| ChromHMM | chr7:151407000..151408400 | Enhancers | Fetal Intestine Large |
| ChromHMM | chr7:151407200..151408000 | Enhancers | Right Ventricle |
| ChromHMM | chr7:151407200..151408200 | Enhancers | Duodenum Mucosa |
| ChromHMM | chr7:151407400..151411400 | Enhancers | Liver |
| ChromHMM | chr7:151407000..151408600 | Genetic enhancers | Fetal Intestine Small |
| ChromHMM | chr7:151406000..151408400 | Heterochromatin | H9 Derived Neuron Cultured Cells |
| ChromHMM | chr7:151359000..151440000 | Quiescent/Low | Primary neutrophils from peripheral blood |
| ChromHMM | chr7:151364400..151452800 | Quiescent/Low | Thymus |
| ChromHMM | chr7:151365600..151452800 | Quiescent/Low | Primary T helper memory cells from peripheral blood 2 |
| ChromHMM | chr7:151377000..151445200 | Quiescent/Low | Primary T helper memory cells from peripheral blood 1 |
| ChromHMM | chr7:151377400..151440000 | Quiescent/Low | Primary T helper naive cells from peripheral blood |
| ChromHMM | chr7:151377600..151409800 | Quiescent/Low | Primary T cells effector/memory enriched from peripheral blood |
| ChromHMM | chr7:151377800..151425000 | Quiescent/Low | Primary T helper 17 cells PMA-I stimulated |
| ChromHMM | chr7:151378200..151409200 | Quiescent/Low | GM12878 Lymphoblastoid Cell Line |
| ChromHMM | chr7:151385600..151409400 | Quiescent/Low | Mesenchymal Stem Cell Derived Chondrocyte Cultured Cells |
| ChromHMM | chr7:151387400..151419000 | Quiescent/Low | Pancreatic Islets |
| ChromHMM | chr7:151388000..151424200 | Quiescent/Low | HUVEC Umbilical Vein Endothelial Primary Cells |
| ChromHMM | chr7:151388000..151424200 | Quiescent/Low | Bone Marrow Derived Cultured Mesenchymal Stem Cells |
| ChromHMM | chr7:151393000..151424600 | Quiescent/Low | Small Intestine |
| ChromHMM | chr7:151394000..151409000 | Quiescent/Low | Foreskin Melanocyte Primary Cells skin03 |
| ChromHMM | chr7:151394400..151422200 | Quiescent/Low | H1 Derived Neuronal Progenitor Cultured Cells |
| ChromHMM | chr7:151394400..151440000 | Quiescent/Low | Primary T CD8+ naive cells from peripheral blood |
| ChromHMM | chr7:151395400..151432400 | Quiescent/Low | ES-UCSF4 Cell Line |
| ChromHMM | chr7:151402400..151458200 | Quiescent/Low | H1 BMP4 Derived Mesendoderm Cultured Cells |
| ChromHMM | chr7:151402800..151409600 | Quiescent/Low | Primary hematopoietic stem cells short term culture |
| ChromHMM | chr7:151402800..151417200 | Quiescent/Low | hESC Derived CD56+ Ectoderm Cultured Cells |
| ChromHMM | chr7:151402800..151426000 | Quiescent/Low | Brain Germinal Matrix |
| ChromHMM | chr7:151402800..151432400 | Quiescent/Low | HUES64 Cell Line |
| ChromHMM | chr7:151403000..151409400 | Quiescent/Low | Brain Hippocampus Middle |
| ChromHMM | chr7:151403000..151414400 | Quiescent/Low | Ganglion Eminence derived primary cultured neurospheres |
| ChromHMM | chr7:151403000..151420600 | Quiescent/Low | Placenta Amnion |
| ChromHMM | chr7:151403200..151409600 | Quiescent/Low | Cortex derived primary cultured neurospheres |
| ChromHMM | chr7:151403200..151417000 | Quiescent/Low | Colon Smooth Muscle |
| ChromHMM | chr7:151403200..151424400 | Quiescent/Low | hESC Derived CD184+ Endoderm Cultured Cells |
| ChromHMM | chr7:151403200..151431000 | Quiescent/Low | iPS-18 Cell Line |
| ChromHMM | chr7:151403400..151409600 | Quiescent/Low | NHDF-Ad Adult Dermal Fibroblast Primary Cells |
| ChromHMM | chr7:151403400..151414600 | Quiescent/Low | Breast variant Human Mammary Epithelial Cells (vHMEC) |
| ChromHMM | chr7:151403400..151414800 | Quiescent/Low | Primary hematopoietic stem cells G-CSF-mobilized Female |
| ChromHMM | chr7:151403600..151422200 | Quiescent/Low | hESC Derived CD56+ Mesoderm Cultured Cells |
| ChromHMM | chr7:151403600..151422800 | Quiescent/Low | NH-A Astrocytes Primary Cells |
| ChromHMM | chr7:151404000..151414800 | Quiescent/Low | HSMM cell derived Skeletal Muscle Myotubes Cell Line |
| ChromHMM | chr7:151404200..151410600 | Quiescent/Low | NHEK-Epidermal Keratinocyte Primary Cells |
| ChromHMM | chr7:151404400..151424200 | Quiescent/Low | Osteoblast Primary Cells |
| ChromHMM | chr7:151404800..151422800 | Quiescent/Low | Fetal Thymus |
| ChromHMM | chr7:151405200..151432400 | Quiescent/Low | H9 Cell Line |
| ChromHMM | chr7:151405200..151432400 | Quiescent/Low | HUES48 Cell Line |
| ChromHMM | chr7:151405800..151412800 | Quiescent/Low | Fetal Muscle Leg |
| ChromHMM | chr7:151405800..151414400 | Quiescent/Low | Muscle Satellite Cultured Cells |
| ChromHMM | chr7:151407200..151422000 | Quiescent/Low | H9 Derived Neuronal Progenitor Cultured Cells |
| ChromHMM | chr7:151407200..151423000 | Quiescent/Low | NHLF Lung Fibroblast Primary Cells |
| ChromHMM | chr7:151407400..151416200 | Quiescent/Low | Mesenchymal Stem Cell Derived Adipocyte Cultured Cells |
| ChromHMM | chr7:151407800..151424400 | Quiescent/Low | ES-WA7 Cell Line |
| ChromHMM | chr7:151407400..151408400 | Repressed PolyComb | Foreskin Fibroblast Primary Cells skin01 |
| ChromHMM | chr7:151407800..151408400 | Repressed PolyComb | Foreskin Fibroblast Primary Cells skin02 |
| ChromHMM | chr7:151405400..151410400 | Strong transcription | Monocytes-CD14+ RO01746 Primary Cells |
| ChromHMM | chr7:151405600..151408000 | Strong transcription | Primary monocytes from peripheral blood |
| ChromHMM | chr7:151399600..151409000 | Weak Repressed PolyComb | Foreskin Melanocyte Primary Cells skin01 |
| ChromHMM | chr7:151402600..151409600 | Weak Repressed PolyComb | H1 Cell Line |
| ChromHMM | chr7:151403200..151409000 | Weak Repressed PolyComb | HUES6 Cell Line |
| ChromHMM | chr7:151403200..151409800 | Weak Repressed PolyComb | ES-I3 Cell Line |
| ChromHMM | chr7:151403400..151411600 | Weak Repressed PolyComb | iPS DF 6.9 Cell Line |
| ChromHMM | chr7:151403600..151418400 | Weak Repressed PolyComb | HepG2 Hepatocellular Carcinoma Cell Line |
| ChromHMM | chr7:151403800..151409400 | Weak Repressed PolyComb | iPS-20b Cell Line |
| ChromHMM | chr7:151403800..151410000 | Weak Repressed PolyComb | iPS-15b Cell Line |
| ChromHMM | chr7:151404000..151409600 | Weak Repressed PolyComb | IMR90 fetal lung fibroblasts Cell Line |
| ChromHMM | chr7:151404000..151415000 | Weak Repressed PolyComb | Dnd41 TCell Leukemia Cell Line |
| ChromHMM | chr7:151404200..151408800 | Weak Repressed PolyComb | Foreskin Keratinocyte Primary Cells skin03 |
| ChromHMM | chr7:151404200..151410000 | Weak Repressed PolyComb | Fetal Adrenal Gland |
| ChromHMM | chr7:151404600..151409800 | Weak Repressed PolyComb | Adipose Derived Mesenchymal Stem Cell Cultured Cells |
| ChromHMM | chr7:151405800..151409200 | Weak Repressed PolyComb | Fetal Muscle Trunk |
| ChromHMM | chr7:151407200..151410200 | Weak Repressed PolyComb | K562 Leukemia Cell Line |
| ChromHMM | chr7:151370800..151442000 | Weak transcription | Primary T regulatory cells from peripheral blood |
| ChromHMM | chr7:151377600..151410800 | Weak transcription | Primary T helper cells PMA-I stimulated |
| ChromHMM | chr7:151394600..151409600 | Weak transcription | Stomach Mucosa |
| ChromHMM | chr7:151400200..151415000 | Weak transcription | Aorta |
| ChromHMM | chr7:151400800..151412800 | Weak transcription | Sigmoid Colon |
| ChromHMM | chr7:151403200..151409600 | Weak transcription | Brain Inferior Temporal Lobe |
| ChromHMM | chr7:151403400..151409400 | Weak transcription | Brain Anterior Caudate |
| ChromHMM | chr7:151403400..151409600 | Weak transcription | Duodenum Smooth Muscle |
| ChromHMM | chr7:151403400..151422800 | Weak transcription | Rectal Smooth Muscle |
| ChromHMM | chr7:151403600..151408200 | Weak transcription | Primary T CD8+ memory cells from peripheral blood |
| ChromHMM | chr7:151403600..151408600 | Weak transcription | HeLa-S3 Cervical Carcinoma Cell Line |
| ChromHMM | chr7:151403600..151440000 | Weak transcription | Primary T cells from peripheral blood |
| ChromHMM | chr7:151403800..151409000 | Weak transcription | Breast Myoepithelial Primary Cells |
| ChromHMM | chr7:151403800..151409400 | Weak transcription | Fetal Brain Female |
| ChromHMM | chr7:151403800..151440000 | Weak transcription | Primary T helper cells from peripheral blood |
| ChromHMM | chr7:151403800..151440200 | Weak transcription | Primary T helper naive cells from peripheral blood |
| ChromHMM | chr7:151404000..151409200 | Weak transcription | HMEC Mammary Epithelial Primary Cells |
| ChromHMM | chr7:151404200..151409200 | Weak transcription | Brain Dorsolateral Prefrontal Cortex |
| ChromHMM | chr7:151404200..151412800 | Weak transcription | Ovary |
| ChromHMM | chr7:151404400..151409000 | Weak transcription | H1 Derived Mesenchymal Stem Cells |
| ChromHMM | chr7:151404400..151409200 | Weak transcription | Lung |
| ChromHMM | chr7:151404400..151409600 | Weak transcription | Brain Cingulate Gyrus |
| ChromHMM | chr7:151404600..151408800 | Weak transcription | A549 EtOH 0.02pct Lung Carcinoma Cell Line |
| ChromHMM | chr7:151404800..151408800 | Weak transcription | Placenta |
| ChromHMM | chr7:151404800..151409000 | Weak transcription | Foreskin Keratinocyte Primary Cells skin02 |
| ChromHMM | chr7:151404800..151416200 | Weak transcription | iPS DF 19.11 Cell Line |
| ChromHMM | chr7:151405800..151413000 | Weak transcription | Colonic Mucosa |
| ChromHMM | chr7:151406200..151409000 | Weak transcription | Left Ventricle |
| ChromHMM | chr7:151406200..151409600 | Weak transcription | Right Atrium |
| ChromHMM | chr7:151406200..151416800 | Weak transcription | Psoas Muscle |
| ChromHMM | chr7:151406400..151409000 | Weak transcription | Fetal Stomach |
| ChromHMM | chr7:151406600..151408400 | Weak transcription | Skeletal Muscle Female |
| ChromHMM | chr7:151406800..151409400 | Weak transcription | Esophagus |
| ChromHMM | chr7:151406800..151409400 | Weak transcription | Primary Natural Killer cells from peripheral blood |
| ChromHMM | chr7:151406800..151422600 | Weak transcription | Primary B cells from cord blood |
| ChromHMM | chr7:151407000..151408800 | Weak transcription | Primary hematopoietic stem cells |
| ChromHMM | chr7:151407000..151409000 | Weak transcription | Fetal Heart |
| ChromHMM | chr7:151407000..151409000 | Weak transcription | Skeletal Muscle Male |
| ChromHMM | chr7:151407000..151409000 | Weak transcription | Spleen |
| ChromHMM | chr7:151407000..151409200 | Weak transcription | HSMM Skeletal Muscle Myoblasts Cell Line |
| ChromHMM | chr7:151407000..151409200 | Weak transcription | Fetal Lung |
| ChromHMM | chr7:151407000..151409400 | Weak transcription | Brain Substantia Nigra |
| ChromHMM | chr7:151407000..151409600 | Weak transcription | Fetal Brain Male |
| ChromHMM | chr7:151407000..151409800 | Weak transcription | Brain Angular Gyrus |
| ChromHMM | chr7:151407000..151409800 | Weak transcription | Rectal Mucosa Donor 31 |
| ChromHMM | chr7:151407000..151409800 | Weak transcription | Primary B cells from peripheral blood |
| ChromHMM | chr7:151407000..151411200 | Weak transcription | H1 BMP4 Derived Trophoblast Cultured Cells |
| ChromHMM | chr7:151407000..151412600 | Weak transcription | Pancreas |
| ChromHMM | chr7:151407000..151413200 | Weak transcription | Rectal Mucosa Donor 29 |
| ChromHMM | chr7:151407000..151415000 | Weak transcription | Primary hematopoietic stem cells G-CSF-mobilized Male |
| ChromHMM | chr7:151407000..151418600 | Weak transcription | Adipose Nuclei |
| ChromHMM | chr7:151407000..151418800 | Weak transcription | Fetal Kidney |
| ChromHMM | chr7:151407000..151440200 | Weak transcription | Primary T cells from cord blood |
| ChromHMM | chr7:151407200..151409000 | Weak transcription | Gastric |
| ChromHMM | chr7:151407200..151409000 | Weak transcription | Stomach Smooth Muscle |
| ChromHMM | chr7:151407600..151409000 | Weak transcription | Primary mononuclear cells from peripheral blood |
